# Who do we follow online? An experimental study on source clarity and social proximity in digital health communication

**DOI:** 10.3389/fpubh.2025.1661328

**Published:** 2025-10-27

**Authors:** Junhui Li, Chunsheng Shi

**Affiliations:** Business School, Harbin Institute of Technology, Harbin, China

**Keywords:** public health promotion, digital health communication, information sources, source clarity, social proximity, health advice compliance, randomized experiment

## Abstract

Public health communication increasingly relies on digital channels where advice is encountered from diverse sources that vary in source clarity (whether the sender is perceived as clearly identifiable) and social proximity (whether the sender is perceived as relationally close). To examine how these sources shape compliance with health advice, we conducted a randomized controlled online experiment (*N* = 810) simulating a social media environment in which each participant viewed one weight-management post attributed to one of eight sources: parents, friends, colleagues, doctors, health influencers, news agencies, Wikipedia, or AI chatbots. We measured intended compliance and four cognitive responses: perceived credibility, psychological reactance, attention, and comprehension. Messages from single specified sources (parents, friends, colleagues, doctors, influencers, news agencies) increased intended compliance by 13–17 percentage points compared with composite diffuse sources (AI chatbots, Wikipedia). Within specified senders, significant others (parents, friends, colleagues) outperformed professional experts (doctors, influencers, news agencies) by 11–16 points. Mediation analyses showed that source clarity operated primarily through enhanced credibility, while social proximity operated through higher credibility and lower reactance; attention and comprehension did not mediate these effects. Subgroup analyses indicated stronger effects among participants with chronic conditions, higher health literacy, or behaviorally aligned daily routines. These findings suggest that, in a networked digital environment, compliance with health advice is influenced less by professional authority or aggregated information, and more by identifiable and socially close sources. The study provides evidence-based guidance for selecting sources and designing messages in public health promotion.

## 1 Introduction

In everyday digital life, people encounter health information across a wide spectrum of contexts—from vaccination reminders and mental health resources to advice on chronic disease management, exercise routines, and nutrition tips. These messages are delivered by diverse sources, including family members, friends, medical professionals, media organizations, online influencers, encyclopedic platforms such as Wikipedia, and emerging tools such as AI chatbots. Their messages reach audiences through multiple venues: short videos on TikTok and longer vlogs on YouTube, explanatory threads on X (formerly Twitter), articles reposted on Facebook, lifestyle tips from influencers on Instagram, search snippets and Wikipedia citations people invoke to bolster a claim, and personalized health advice circulating in WhatsApp and Messenger groups. Public surveys show that many people welcome these inputs: in the United States, 65% of adults report that the internet and social media help them improve their health behaviors (1). Yet the multiplicity of sources also creates uncertainty, as some are easily identifiable while others are diffuse, and some feel socially close while others are distant or impersonal. For public health promotion and education, this raises a central communication challenge: in today’s networked digital environment, who is trusted as a health source, and on what basis, remains insufficiently understood.

Health communication research has long shown that audiences rely on cues about the source when deciding whether to accept advice. Classic studies emphasized indicators such as expertise and trustworthiness (2–4) and the influence of interpersonal ties (5). More recent work in digital settings has examined how visible signals (such as author labels, professional design, and social endorsement) shape credibility (6–8) and has broadened what counts as a “source,” to include institutions, algorithms, and collective voices (9). In highly networked, repost-driven environments, people rarely trace messages back to their origin: a Wikipedia extract or a Chatbot-generated answer may be taken at face value, while reposted infographics or links often arrive via family, friends, or colleagues. In these settings, credibility judgments are often made when cues about who is speaking are incomplete, indirect, or obscured.

Against this backdrop, three considerations motivate our study and extend prior work. First, much existing research examines a single source type or a narrow contrast (e.g., experts vs. peers). Such designs provide important insights (10–13), but they do not capture the relative influence of multiple commonplace sources when evaluated side by side under shared conditions. Second, prior findings are often context dependent and fragmented. During infectious-disease crises, expert voices are typically persuasive and authoritative (14), whereas in routine self-management, peers and platform-level sources have sometimes been found to exert equal or greater influence (15). Because studies vary in topic, platform, and measurement, their conclusions are not directly comparable, leaving open whether divergent results reflect differences among sources themselves or the contexts in which they were studied. Third, leading theoretical perspectives make competing predictions about how source effects operate. Some suggest that sources signaling expertise and trustworthiness are perceived as more credible and therefore increase compliance, while others emphasize that these same sources may be interpreted as overt persuasion attempts, triggering resistance and lowering compliance (2, 3). These contradictory predictions highlight the need to examine alternative mechanisms in parallel, both theoretically and empirically, within a unified framework of source credibility for public health communication.

To address these issues, we classify health information sources by two perceptual dimensions that capture how audiences typically evaluate them in digitally mediated communication contexts. The first is *source clarity*, distinguishing senders that are clearly identifiable (e.g., a doctor’s post, a friend’s forward) from those that appear diffuse or aggregated (e.g., an encyclopedia entry, a chatbot output). The second is *social proximity*, distinguishing relationally close senders (e.g., family, friends, colleagues) from more distant or institutional ones (e.g., doctors, health influencers, news agencies). Cross-classifying these dimensions yields eight everyday exemplars that represent the range of sources people encounter online. Guided by this classification, we ask: *How do health information sources, which* var*y in clarity and social proximity, shape individuals’ intention to comply with online health advice?*

To examine this question in a concrete yet generalizable setting, we implement a unified experimental design in the domain of weight management, focusing on everyday advice about diet and exercise. This choice rests on three considerations. First, weight management is one of the most common topics in digital health communication, widely circulated across platforms (1, 16, 17). Second, it is personally salient for broad adult populations, as diet and exercise routines are integral to daily self-care. Third, it is characterized by contested claims (such as intermittent fasting or exercise timing) where advice of varying credibility circulates (15, 16, 18). These features make weight management a suitable testbed: it allows us to hold health content and settings constant while varying perceived source, thereby enabling a clearer comparison across sources while speaking to broader challenges in digital public health communication.

The remainder of the article is organized as follows. Section 2 reviews the relevant health communication literature and develops hypotheses regarding the effects of source clarity and social proximity, as well as the underlying cognitive mechanisms that drive these effects. Section 3 describes the experimental design and data. Section 4 presents results. Section 5 discusses key findings, theoretical contributions, practical implications, and limitations. Section 6 concludes.

## 2 Theoretical background and hypotheses development

This section outlines the conceptual foundation for our study. We begin by reviewing communication research on how audiences perceive and classify health information sources in digital environments. Building on this, we discuss two theoretical perspectives, Source Credibility Theory and Persuasion Knowledge Theory, and clarify the cognitive process an individual travels to make decisions upon the information they receive. Then we integrate these insights to develop hypotheses on how information sources influence compliance with health advice.

### 2.1 Online health information sources and classification

Contemporary public health communication can be understood through two perceptual dimensions that shape how audiences evaluate messages at the point of encounter: *source clarity* and *social proximity*. Source clarity concerns whether the speaker is readily identifiable, while *social proximity* concerns the perceived relational closeness between sender and receiver. These distinctions are particularly salient in repost-driven environments, where audiences often form judgments without tracking content back to its original author (19, 20).

*Source clarity* separates clearly identifiable senders from composite/diffuse ones. Physicians typically post under their own names and credentials, making the speaker explicit and accountable for the message. News agencies publish under a recognizable masthead and byline, so readers often attribute the message to a specific institution with editorial standards. Health influencers communicate through stable handles and channel identities that persist across posts, allowing attribution to a particular persona. Parents are recognized as known family members, so their messages clearly come from a specific individual. Friends appear with their names in the feed or message header, leaving little ambiguity about who is speaking. Colleagues are also identifiable through their personal profiles within the recipient’s social or work network (2–5, 11, 20–23).

By contrast, Wikipedia presents content under a collective platform label that aggregates many contributors. Readers typically experience the text as coming from “Wikipedia” rather than a named author (10). Although such entries include references and edit histories, few users trace them, so authorship remains diffuse at the point of use. AI chatbots operate in a similar but technologically mediated way. They return synthesized answers drawn from large text corpora, producing responses that appear authoritative yet lack a visible human byline (8). Recent studies show that these conversational agents are becoming common channels for health advice (24). They offer clear advantages, such as speed, personalization, and low cost (25), but also raise concerns about credibility, trust, and engagement (26, 27). In both cases, users interact with information that feels impersonal and collectively produced.

These patterns align directly with our focus on source clarity. When a health information source is clearly identifiable, audiences can assign accountability and infer expertise; when the sender appears aggregated or synthesized, responsibility is harder to locate, which may in turn affect whether advice is judged trustworthy and actionable (2, 4, 6, 8, 19, 28).

*Social proximity* further differentiates among identifiable sources by perceived closeness. In our usage, social proximity applies only to clearly identifiable human or institutional senders; composite sources such as Wikipedia or AI chatbots do not present relational entities and are therefore classified by clarity alone. Parents, friends, and colleagues typically represent close or moderately close ties, whose advice is often construed as benevolent and personally relevant, which may facilitate influence via trust, reciprocity, and social reinforcement (5, 11, 21, 22). Physicians, news agencies, and health influencers are likewise identifiable but generally more distant, deriving influence from professional expertise, institutional legitimacy, or parasocial connection rather than reciprocity (2, 4, 6–8, 20, 23). Social proximity matters: close ties can reduce resistance in everyday self-management, while professional or institutional actors may heighten perceived competence. These are distinct pathways through which sources may influence compliance (2, 5, 11, 21, 22, 29, 30).

To anchor our classification, we clarify what we mean by “source.” We follow Sundar and Nass (19) to differentiate two senses of sources: an *ontological* sense, the original creator or institutional author, and a *perceived* sense, the actor the audience experiences as speaking at the moment of encounter. In digitally networked, repost-driven settings, audiences often meet content as forwards, outlet-labeled snippets, or system-generated answers, so credibility judgments are formed with reference to the perceived actor rather than the originator (19, 23). Therefore, this study adopts the perceived-source perspective and classifies health information sources as they appear at encounter.

### 2.2 Theoretical foundations for information source effects

Understanding how people respond to online health advice requires not only identifying the perceived sources but also considering the cognitive processes that connect those perceptions to subsequent decisions. Two frameworks are particularly relevant for this purpose: Source Credibility Theory, which explains how audiences infer qualities such as expertise or trustworthiness from senders, and the Persuasion Knowledge Model, which highlights how people may resist when they perceive persuasive intent. These perspectives suggest complementary yet contrasting pathways through which source perceptions may shape compliance with health advice.

Source Credibility Theory has its origins in classic communication research showing that judgments of *expertise* and *trustworthiness* strongly influence whether messages are accepted (2). Later work added *attractiveness*, emphasizing that perceptions of empathy, likability, and interpersonal appeal also matter in health communication (3). These three dimensions are often treated as facets of *perceived credibility* (4, 31). Importantly, credibility is not a fixed attribute of the sender but a perception constructed by the receiver (7). Such perceptions differ across settings: a clinician may be viewed as highly expert but less relatable, while a friend may seem caring but less competent. In digital health communication, where sources vary in both clarity and social proximity, credibility offers a primary lens for understanding why some messages elicit stronger compliance than others.

The Persuasion Knowledge Model (29) offers a complementary perspective by focusing on how people interpret persuasive intent. As individuals gain experience with diverse communication settings, they develop *persuasion knowledge*, that is, generalized expectations about others’ motives and tactics. When a message is perceived as strategically motivated, recipients may experience *psychological reactance* (32), a defensive state marked by irritation, skepticism, or feelings of being manipulated. This resistance can manifest both *cognitively* by doubting the accuracy of a message or *behaviorally* by rejecting the advice (30, 33). In digital environments where professional, peer, and algorithmic voices coexist, overtly directive or authoritative messages may heighten reactance, whereas socially close or seemingly neutral sources may reduce it.

Beyond credibility and reactance, theories of information processing highlight the basic conditions that enable such evaluations. Dual-process models such as the *Elaboration Likelihood Model* (34) emphasize that *attention* to and *comprehension* of message content are prerequisites for forming judgments. In digital health communication, where information is often encountered quickly, such processing may be limited, yet these factors remain useful indicators of cognitive engagement (20, 35). Although not central to our theoretical framework, attention and comprehension may help capture the cognitive conditions under which source effects unfold.

### 2.3 Hypotheses development

**Figure 1:**
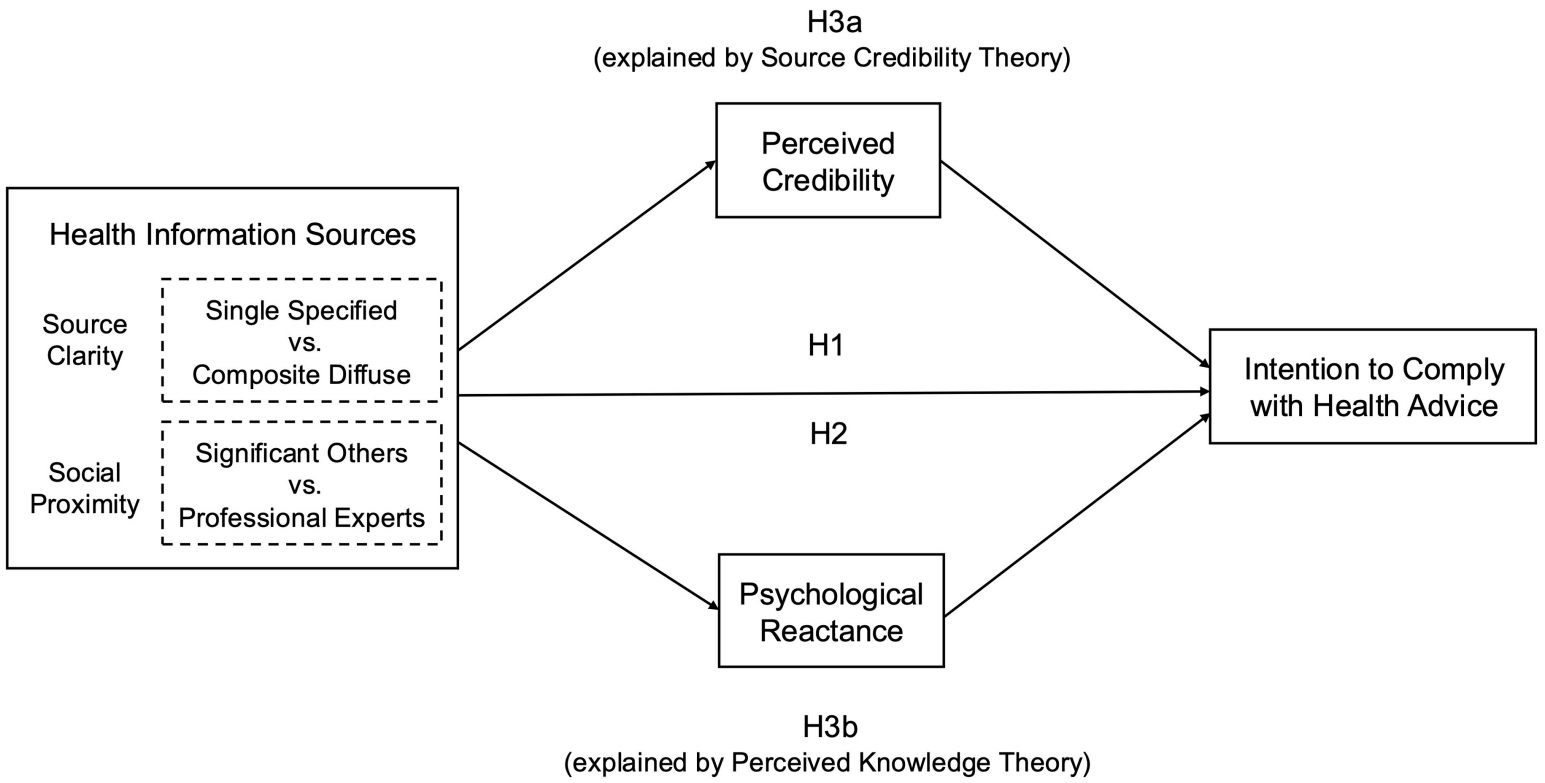
A theoretical framework of information sources and compliance with health advice.

Building on the theoretical perspectives reviewed above, we develop hypotheses that address the direct effects of source clarity and social proximity on compliance with health advice (H1 and H2) and the cognitive pathways through which these influences may occur (H3). Figure 1 illustrates the conceptual framework that encompasses the key constructs and hypotheses.

#### 2.3.1 The effect of source clarity

In everyday digital environments, health advice may come from a clearly identifiable sender or from an aggregated, diffuse source. A physician posting an infographic represents a single specified source whose credentials are visible, whereas a chatbot generating a diet plan presents a composite output with hidden authorship. Source Credibility Theory (SCT) emphasizes that specified sources are more persuasive because audiences can attribute expertise and trustworthiness to an identifiable individual or institution (2–4). By contrast, the Persuasion Knowledge Model (PKM) highlights that specified sources may also activate resistance if their persuasive intent is obvious (29, 30). In such cases, a chatbot’s response could be accepted as neutral and less manipulative because it lacks a visible persuader. These competing perspectives lead to the following hypotheses:

*H1a (SCT prediction)*: Single specified sources are more likely to increase compliance with health advice compared to composite diffuse sources.

*H1b (PKM prediction)*: Composite diffuse sources are more likely to increase compliance with health advice compared to single specified sources.

#### 2.3.2 The effect of social proximity

Even when a source is specified, audiences draw different inferences depending on whether the sender is a close tie or a professional expert. A diet tip shared by a friend in a group chat and a column written by a journalist in a newspaper are both specified senders, yet they invite different heuristics. Professional experts may hold an advantage because they embody knowledge and competence, qualities especially valued when health choices carry real consequences (31). Yet expertise is not always persuasive. When advice from professionals or institutions is perceived as directive, audiences may feel pressured and resist. Messages from significant others, by contrast, may be received as authentic and caring, even if they lack formal credentials (11). A young adult might dismiss a nutritionist’s advice as “preachy” but adopt a workout tip from a friend because it feels personally relevant and less like persuasion. These tensions motivate our second set of hypotheses:

*H2a (SCT prediction)*: Professional experts are more likely to increase compliance with health advice compared to significant others.

*H2b (PKM prediction)*: Significant others are more likely to increase compliance with health advice compared to professional experts.

#### 2.3.3 The mediating role of credibility and reactance

Both SCT and PKM further suggest that source effects may operate through cognitive mediators, but in opposite directions.

SCT highlights a positive pathway: Specified and expert sources can foster compliance because they heighten perceptions of credibility, including perceived expertise, trustworthiness, and attractiveness (3, 4, 20). Source clarity strengthens credibility by making it easier to attribute expertise and accountability to a visible sender; for example, a physician posting an infographic is perceived as knowledgeable and responsible in a way that a chatbot summary is not. Social proximity can also reinforce credibility through different dimensions: advice from a parent or friend may be trusted as benevolent and caring, enhancing trustworthiness and likability even when formal expertise is absent.

PKM highlights a negative pathway: When persuasive intent is salient, audiences may feel manipulated and resist the message (30, 32, 33). Source clarity can heighten this perception if the sender appears overtly strategic; for instance, a physician’s prescriptive message may feel controlling, or a health influencer’s post may be seen as pushing products or medications for commercial gains. Social proximity, by contrast, often dampens reactance: the same recommendation framed by a friend or colleague may feel more authentic and less likely to trigger resistance.

These perspectives suggest that credibility and reactance can both serve as mediating pathways through which source clarity and social proximity influence compliance. Our study, therefore, tests both mechanisms in parallel:

*H3a*: The effect of single specified sources, particularly professional experts, on compliance is mediated by *credibility*: higher perceived credibility is more likely to increase compliance.

*H3b*: The effect of single specified sources, particularly professional experts, on compliance is mediated by *reactance*: higher reactance is more likely to reduce compliance.

Taken together, these hypotheses reflect theoretical tensions between SCT and PKM (see Table 1 for a summary). Clarifying how these mechanisms operate requires empirical evidence, which we provide by directly comparing multiple source types in a unified experimental setting.

**Table 1:** Overview of hypotheses and theoretical predictions.

Hypothesis	Source dimension	Predictions from the source credibility theory	Predictions from the persuasion knowledge theory
H1a/H1b	Source clarity (single specified vs. composite diffuse)	Single specified sources more persuasive due to identifiable credibility cues (expertise, trustworthiness)	Composite sources may elicit more compliance because diffuse authorship reduces perceived persuasive intent
H2a/H2b	Social proximity (significant others vs. professional experts)	Professional experts more persuasive due to perceived knowledge and competence	Significant others more persuasive because they are seen as authentic and less manipulative
H3a/H3b	Mediating pathways	Source effects transmitted *positively* through credibility (expertise, trustworthiness, attractiveness)	Source effects transmitted *negatively* through psychological reactance (perceived manipulation, resistance)

## 3 Methods

### 3.1 Context

The experiment was fielded in an online environment designed to resemble a scrolling social media feed, reflecting how health information is typically encountered in contemporary public health communication (36). Participants read short posts on weight management, a domain that appears frequently in online searches, social media content, and public health campaigns (1).

Within the weight management domain, we focused on two widely discussed topics, namely *intermittent fasting* and *exercise timing*, because they exemplify familiar yet contested forms of everyday health advice. Recent systematic reviews identify intermittent fasting as one of the most empirically examined and publicly popular dietary strategies, having gained substantial attention as a behavioral approach to weight control (37). Similarly, whether exercise is more effective in the morning or evening remains an open question, debated in both academic research and popular media, with evidence to date mixed and inconclusive (38). The familiarity of these topics allows participants to evaluate the advice without needing specialized medical knowledge, ensuring that any variation in compliance reflects the source manipulation rather than differences in topic complexity or novelty.

All materials were created in Qualtrics and delivered via Prolific. Prolific provides a large, demographically varied participant pool, enabling random assignment to experimental conditions while maintaining data quality and attentiveness (39, 40). Using this platform allowed us to recruit participants who reflect the diversity of the general population and to minimize attrition biases typical of field recruitment. It also ensured that the experimental setting resembled how people commonly encounter health information online, through brief and decontextualized posts while browsing digital feeds.

### 3.2 Experimental design and procedure

#### 3.2.1 Consent and background survey

**Figure 2:**
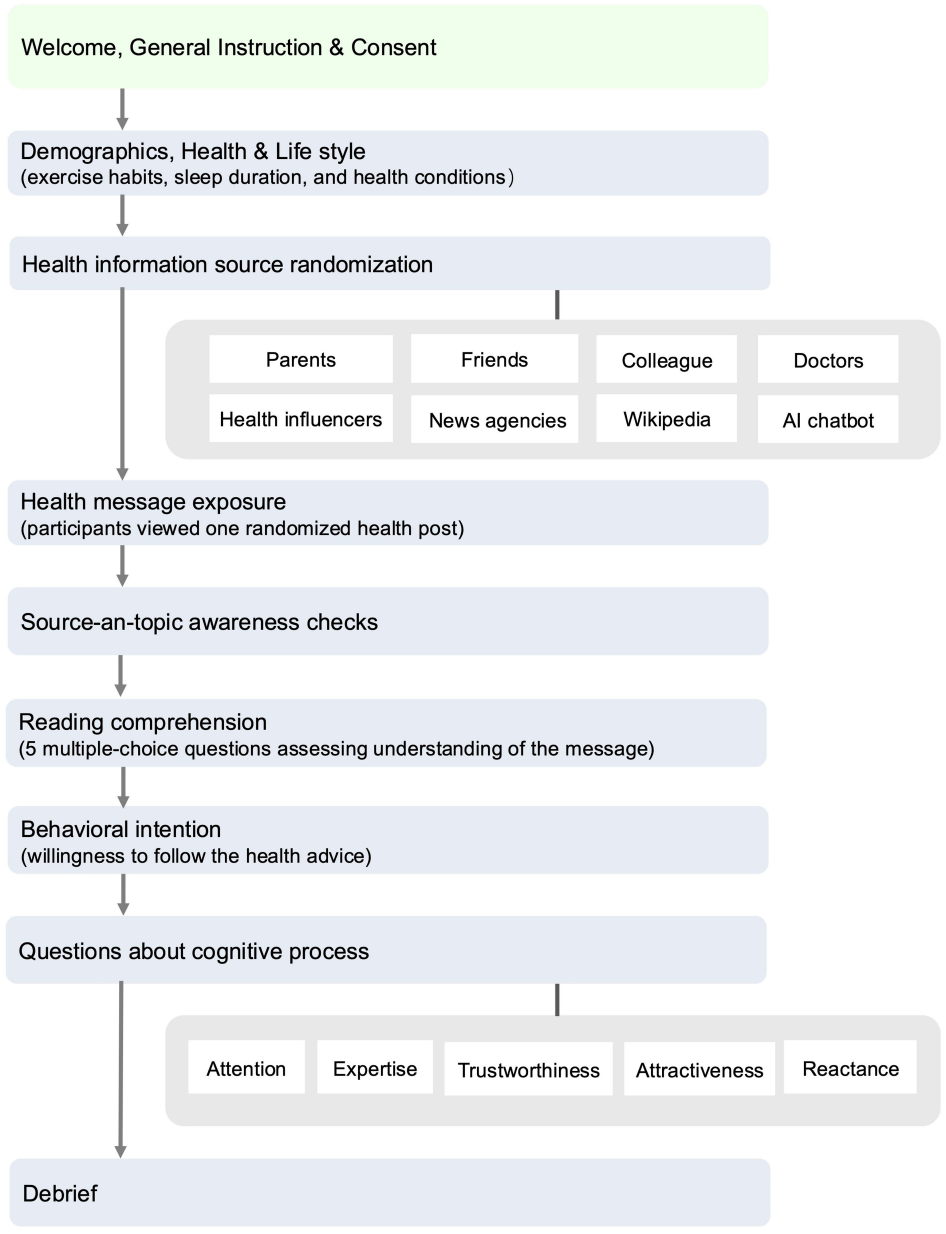
Experimental procedure.

At the beginning of the online experimental study (see Figure 2 for the full procedure), participants read an informed consent form describing the nature of the experiment and their rights. The form explained that the health posts they would read were adapted from everyday online content rather than verified medical advice, and that the purpose of the study was to examine how people respond to such information. Participants were reminded that any decision to follow or not follow the advice was entirely their own choice. Only those who provided explicit consent proceeded to the experiment, which began with a short background questionnaire. This questionnaire collected demographic characteristics (age, gender, education), physical measures (height and weight to compute body mass index), health conditions, self-assessed health awareness and literacy, and routine diet and exercise habits. These variables are used as covariates in the analysis, with their definitions and measures reported in Supplementary Table S2.

#### 3.2.2 Source assignment

After the survey, participants were randomly assigned to one of eight source labels. Six labels represented single specified senders, including three significant-other roles (parent, friend, work colleague) and three professional roles (doctor, health influencer, news agency). The remaining two labels represented composite, diffuse sources, described as Wikipedia or an AI-powered chatbot. The participant saw a message such as: “*You will soon read a health message. While reading, imagine the message is from [assigned source]*.” For example, the assigned source might be a health influencer encountered on social media. Table 2 details the source stimuli, and Supplementary Tables S3–S5 report covariate balance checks for the pooled source contrasts (*t*-tests) and for all eight groups (Pearson χ^2^ tests); none show statistically significant differences, indicating that randomized source assignment was well-performed.

**Table 2:** All experimental conditions for information sources.

Categories		Sources	Stimuli
Single specified sources	Significant others	Parent	While you are reading, imagine the message is from your *parents*
		Friend	While you are reading, imagine the message is from one of your *friends*
		Colleague	While you are reading, imagine the message is from one of your *colleagues* at work
	Professional experts	Doctor	While you are reading, imagine the message is from one of the *doctors* you met before
		Health influencer	While you are reading, imagine the message is from one of the *health influencers* you saw on social media
		News agency	While you are reading, imagine the message is from one mainstream *news agency* in your country
Composite diffuse sources		Wikipedia	While you are reading, imagine the message is from *Wikipedia*
		AI Chatbot	While you are reading, imagine the message is from an *AI chatbot*

Immediately following the exposure to the stimuli, we provide brief descriptions of Wikipedia and AI chatbots to ensure that participants assigned to these conditions have a clear understanding of these sources, regardless of their prior familiarity: (1) Wikipedia: If you have never heard of or used Wikipedia, please refer to the following description: “Wikipedia is a free online encyclopedia, created and edited by volunteers around the world.” (2) AI Chatbot: If you have never heard of or used an AI chatbot, please refer to the following description: “An AI chatbot is a software application that uses natural language processing techniques to interact with users. When a user types a message, the chatbot analyzes the words and phrases to understand the user’s intent, searches its database for relevant information, and returns the most appropriate response”.

Our grouping of these eight exemplars follows the conceptual distinctions outlined in Section 2.1. Single specified sources are those whose identity is perceived as clear and embodied in a person or institution, further subdivided into significant others (parent, friend, colleague) and professional experts (doctor, health influencer, news agency). This subdivision reflects evidence that audiences apply different heuristics depending on whether advice comes from relationally close ties (trusted as altruistic and personally relevant) or credentialed actors (evaluated on expertise and institutional legitimacy). Composite diffuse sources (Wikipedia, AI chatbot) represent advice encountered as aggregated and de-personalized, where authorship is opaque at the point of use. Although Wikipedia and chatbots differ technically, both are experienced as synthesized outputs without a single identifiable author, which justifies treating them together in this design.

#### 3.2.3 Health messages

After source assignment, participants viewed a simulated social media post with a headline and fewer than one hundred words of text. Four posts were created on weight management, covering two common themes: a diet pair titled “*16:8 Fasting is Good for Your Health*” and “*16:8 Fasting is Not Good for Your Health*,” and an exercise pair titled “*Morning Exercise is Good for Your Health*” and “*Evening Exercise is Not Good for Your Health*” (see Supplementary Table S6). These topics were chosen because they are familiar, frequently discussed online, and often presented with conflicting claims. This design allowed us to reproduce the contested and polarized nature of digital health content. All message texts were adapted from reputable health and government websites^1^^,^^2^ and were matched for length, tone, and readability. Random assignment ensured that each participant viewed one of the four posts in the context of their assigned source. As noted in the consent form (see Section 3.2.1) and reiterated in the debriefing (see Section 3.2.6), participants were informed that these materials simulated everyday online content and were not verified medical advice.

#### 3.2.4 Source-and-topic awareness checks

After participants were assigned to a source label and completed reading the health message, two short questions were used to assess whether they were aware of the condition to which they had been assigned. The first asked participants to indicate who the health message was from, and the second asked them to identify the specific post they had just read. The purpose of these awareness checks was to confirm that participants noticed their assigned condition and the content they viewed. These questions were not intended to test whether the manipulations successfully altered perceived source clarity or social proximity—a notable limitation acknowledged in Section 5.4. We calculated the proportion of participants who answered each question correctly, reported as pass rates in Supplementary Table S7. All participants were retained in the main analysis to preserve random assignment and external validity, and results were similar when restricting the sample to those who passed both checks (see Supplementary Table S8).

#### 3.2.5 Post-exposure measures

Following message exposure and the manipulation checks, participants completed several questions assessing their comprehension of the health message, prior exposure to similar advice, and intention to comply with the advice. They also rated their attention, credibility perceptions, and psychological reactance. The survey items and variable coding are described in §3.3 and summarized in Table 3.

**Table 3:** Key variables, definitions, and measurement.

Variable	Description	Measurement basis
Information source	Dichotomized treatment variable indicating the attributed sender of the health advice	Eight labels: parent, friend, work colleague, doctor, health influencer, news agency, Wikipedia, AI chatbot. Analysis contrasts: (i) Single specified (=1) vs. Composite diffuse (=0); (ii) Within single source, Significant others (=1) vs. Professionals (=0)
Compliance intention	Behavioral tendency outcome: likelihood of following the health advice	Participants indicated compliance intention on a four-point ordinal scale: (1) “*I will not take the advice*”; (2) “*I will consider the advice but lean toward not taking it*”; (3) “*I will consider the advice and lean toward taking it*”; and (4) “*I will take the advice*.” For the main OLS and logit analyses, we dichotomized the measure by combining responses (1–2) versus (3–4), coding willingness to comply as 1 and non-compliance as 0. As robustness checks, we also estimated ordered logit models using the full four-point scale, comparing the likelihood of being in higher compliance categories (≥3) versus lower categories (<3)
Perceived credibility	Audience judgments of the sender’s credibility	Three subscales: expertise (e.g., “*The source is knowledgeable about the topic*”), trustworthiness (e.g., “*The source is honest*”), attractiveness (e.g., “*The source is attractive*”). Seven-point agreement ratings (−3 to 3); combined as latent construct in structural equation mediation analysis
Psychological reactance	Defensive response to perceived persuasive intent	Six items adapted from McCroskey et al. (3), Pornpitakpan (4), and Hong and Faedda (32); e.g., “*The health message tries to manipulate me*.” Responses on a seven-point scale (−3 to 3)
Attention	Cognitive engagement with the health message	Four seven-point agreement ratings between −3 and 3 (e.g., “*I paid close attention to the health information*”) adapted from Kang and Sundar (35), measuring focus and interest while reading
Comprehension	Objective understanding of the health message	Five multiple-choice factual questions (e.g., “*What benefit does the 16:8 diet offer in terms of calorie intake*?” for participants assigned to “16:8 Fasting is Good for Your Health”) tailored to the assigned health message. Score = proportion correct (0–1)

For the baseline analysis of the direct information source effects, each construct was operationalized as the average of its constituent items.

#### 3.2.6 Debriefing and ethics

At the end of the study, participants viewed a debriefing screen clarifying that the materials were designed to simulate everyday online content and encouraged participants to consult health professionals for personal guidance: “*We do not verify the correctness of the health information presented. Our goal is to understand how individuals process and respond to health messages similar to those encountered in daily life. For any health advice on topics such as weight loss, we encourage you to consult with a qualified health professional.*” The study protocol, including consent and debriefing, was reviewed and approved by the Research Ethics Committee of the Business School, Harbin Institute of Technology (Ref No. SX-LLSC-2025-005), ensuring that participants were ethically protected and not misled.

### 3.3 Key variables and measurement

The independent variable is the *source* to which each health message was attributed. Participants were randomly assigned to one of eight labels: three significant others (parent, friend, work colleague), three professional experts (doctor, health influencer, news agency), and two composite senders (Wikipedia, AI chatbot). For analysis, we constructed two source contrasts. The first distinguishes single specified from composite diffuse sources, coded as 1 for parent, friend, colleague, doctor, influencer, or news agency, and 0 for Wikipedia or chatbot. The second is restricted to the single-source subsample and contrasts significant others with professional experts, coded as 1 for parent, friend, or colleague, and 0 for doctor, influencer, or news agency. This operationalization follows the conceptual distinctions introduced in Section 2.1 and allows us to test the influence of both source clarity and social proximity.

The dependent variable is the *intention to comply with the health advice*. Participants reported their likelihood of following the message by selecting one of four statements ranging from refusal to adoption (e.g., “I will not take the advice,” “I will take the advice”). For the main analysis, this measure was dichotomized into an indicator of compliance, coded 1 for intending to comply and 0 otherwise. This dichotomization enables percentage-point interpretation of effect sizes, although robustness checks with ordered logit models confirmed that the results are consistent when the full ordinal measure is retained.

To examine potential cognitive pathways, we measured four constructs anchored in established instruments. *Perceived credibility* was assessed with three sub-dimensions, including expertise, trustworthiness, and attractiveness, which draw on the classic source credibility scales of McCroskey and Teven (3) and subsequent validations (4, 23). An example item is “The source is knowledgeable about the topic.” *Psychological reactance*, reflecting defensive responses to persuasive intent, was measured with six items adapted from Hong and Faedda (32), such as “The health message tries to manipulate me.” *Attention* to the health message, defined as the degree of engagement during exposure, was measured with items adapted from Kang and Sundar (35), including “I paid close attention to the health information.” All these cognitive constructs were collected on seven-point agreement scales and normalized to a range from −3 to +3 to better capture both positive and negative responses. For each construct, we computed the mean of its constituent items to obtain a single composite score, which was then used in the regression and mediation analyses. Reliability analyses showed that all multi-item scales demonstrated high internal consistency: attention (Cronbach’s α = 0.82), expertise (α = 0.95), trustworthiness (α = 0.91), attractiveness (α = 0.91), and reactance (α = 0.89). These values exceed the conventional threshold of 0.70, indicating satisfactory reliability (41). Finally, *comprehension* was operationalized as objective understanding of message content, assessed through five multiple-choice questions about the assigned post, with scores ranging from 0 (no correct answers) to 1 (all five correct).

Table 3 summarizes the operationalization of the key variables, and Table 4 presents their descriptive statistics. Full details of covariates, survey items, and coding are provided in Supplementary Tables S1, S2.

**Table 4:** Summary statistics.

Variable	(1)	(2)	(3)	(4)
	Mean	Standard deviation	Min	Max
Gender	1.407	0.492	1.000	2.000
Age	29.074	8.935	18.000	62.000
Education	4.327	1.388	1.000	7.000
Income	3.025	2.535	1.000	12.000
Income (perceived)	2.167	0.748	1.000	4.000
Health info frequency	2.037	1.564	0.000	6.000
Timing for breakfast	8.914	2.099	0.000	17.000
Timing for lunch	13.722	2.263	0.000	22.000
Timing for dinner	19.648	2.225	3.000	24.000
Timing for exercise	15.086	5.038	1.000	23.000
Duration of sleeping (hours)	7.143	1.521	0.000	12.500
Duration of sitting for work (hours)	6.312	2.847	0.000	15.000
Duration of sitting for leisure (hours)	4.131	2.530	0.500	13.400
Duration of exercise (hours)	1.193	0.797	0.000	5.000
Alcohol (0–4)	1.377	1.112	0.000	4.000
Smoke (0–1)	0.228	0.420	0.000	1.000
Medical conditions (0–1)	0.259	0.438	0.000	1.000
Self-perceived healthiness	0.105	1.617	−3.000	3.000
Self-health concern	0.525	1.553	−3.000	3.000
Self-body shame	0.741	1.647	−3.000	3.000
Similar advice (heard)	0.556	0.497	0.000	1.000
Similar advice (followed)	0.489	0.500	0.000	1.000
Perceived expertise	0.792	1.460	−3.000	3.000
Perceived trustworthiness	0.868	1.332	−3.000	3.000
Perceived attraction	0.728	1.276	−3.000	3.000
Reactance	−1.444	1.181	−3.000	2.333
Attention to health message	2.060	0.852	−0.250	3.000
Comprehension accuracy	0.863	0.177	0.400	1.000
Compliance intention	0.611	0.488	0.000	1.000

### 3.4 Empirical models

To test whether information source influences compliance with health advice (H1 and H2), we regressed compliance intention on the experimental treatments, operationalized as two source contrasts. The first compared single specified sources with composite diffuse sources, and the second compared significant others with professional experts within the single-source group.

Our primary specification was a linear probability model (LPM) estimated by ordinary least squares (OLS), which reports percentage-point changes in compliance, making them straightforward for public health audiences to interpret. Although multivariate ANCOVA is sometimes used to analyze public health experiments, its assumption of normally distributed outcomes is not met here. The LPM offers a transparent linear specification that facilitates comparability across nested models.

We first estimated models with only the treatment indicators, as random assignment already provides unbiased estimates of source effects. We then added covariates for demographics (age, gender, education, income), routine behaviors (timing and duration of daily exercise, sitting, eating, sleeping, alcohol consumption, and smoking), and health status (self-perceived health, health concerns, medical conditions). While not required for causal identification, these covariates improved the estimation precision and allowed us to assess the stability of treatment effects across specifications.

In addition to the LPM, we also estimated logistic regressions for the binary compliance indicator and ordered logistic regressions for the original four-point scale. These nonlinear models are conventional choices for categorical outcomes and served as cross-validation of the results across different estimators. For comparability, results are presented as average marginal effects.

To examine whether source effects operate through cognitive pathways (H3), we estimated structural equation models (SEM) with bootstrapped standard errors (100 replications). Compliance was modeled as the outcome, regressed on the two source contrasts and four mediators: perceived credibility, psychological reactance, attention, and comprehension. Perceived credibility was modeled as a latent construct with expertise, trustworthiness, and attractiveness specified as reflective indicators, so that the latent factor captures their shared variance. Reactance was modeled as a composite scale formed from its constituent items. Attention and comprehension were included as observed mediators in parallel, allowing us to test whether the amount of focus participants devoted to the message or their understanding of its content carried source effects to compliance. This SEM framework enables a formal assessment of competing predictions from Source Credibility Theory and the Persuasion Knowledge Model.

## 4 Results

### 4.1 Main effects on health advice compliance

Tables 5, 6 report the effects of information sources on compliance with health advice. Messages attributed to a single specified source increased compliance intention by 0.132 (*p* < 0.01) compared to composite diffuse senders. Adding demographics, routine behaviors, and health covariates increased the coefficient to 0.165 (*p* < 0.01). Although coefficients rose after adding controls, we used *Z*-tests to check whether coefficients differed across specifications; this test provides a formal way to assess whether the inclusion of covariates significantly altered the treatment effect estimates (42). All shifts were not significant (all *p* > 0.30). For example, the difference between the lowest and highest estimates (0.108 vs. 0.165) yielded | *Z* | = 0.98, *p* = 0.33, indicating stability across specifications. Within single specified sources, messages from significant others produced higher compliance than those from professional experts. Across models with controls, the effect size ranged from 0.150 to 0.161 (all *p* < 0.01). *Z*-tests again show no significant differences across specifications (| *Z* | = 0.90, *p* = 0.37).

**Table 5:** OLS estimates on the effects of single source (vs. composite source) on the intention to comply with the health advice.

	DV: advice compliance			
	(1)	(2)	(3)	(4)
	No control	+ Demographics	+ Behavior	+ Health
Single source (vs. composite source)	0.132*** (0.040)	0.108*** (0.041)	0.159*** (0.042)	0.165*** (0.041)
BMI		0.010*** (0.003)	0.008** (0.004)	0.011*** (0.004)
Gender		0.145*** (0.033)	0.133*** (0.037)	0.125*** (0.037)
Age		0.012*** (0.002)	0.014*** (0.002)	0.014*** (0.002)
Education		−0.001 (0.013)	−0.002 (0.015)	−0.006 (0.016)
Income		−0.038*** (0.008)	−0.033*** (0.025)	−0.035*** (0.008)
Income (perceived)		0.085*** (0.025)	0.114*** (0.027)	0.114*** (0.027)
Health info frequency			−0.011 (0.011)	−0.010 (0.012)
Timing for breakfast			0.018** (0.009)	0.018** (0.009)
Timing for lunch			−0.012 (0.008)	−0.015* (0.008)
Timing for dinner			−0.013 (0.012)	−0.006 (0.013)
Timing for exercise			0.000 (0.004)	−0.001 (0.004)
Duration of sleeping			0.001 (0.011)	−0.001 (0.012)
Duration of sitting for work			−0.002 (0.006)	0.000 (0.006)
Duration of sitting for leisure			0.002 (0.007)	0.002 (0.007)
Duration of exercise			0.120*** (0.020)	0.112*** (0.020)
Alcohol			−0.087*** (0.016)	−0.087*** (0.016)
Smoke			0.141*** (0.043)	0.162*** (0.044)
Self-perceived healthiness				0.021* (0.011)
Self-health concern				−0.015 (0.012)
Self-body shame				0.014 (0.012)
Medical conditions				−0.054 (0.042)
Demographics	NO	YES	YES	YES
Routine behaviors	NO	NO	YES	YES
Health	NO	NO	NO	YES
Constant	0.512*** (0.035)	−0.337*** (0.116)	−0.230 (0.265)	−0.361 (0.265)
Observations	810	810	810	810
R-squared	0.014	0.112	0.201	0.210

Robust standard errors in parentheses. ***p < 0.01, **p < 0.05, *p < 0.1.

**Table 6:** OLS estimates on the effects of significant other (vs. professional expert) on the intention to comply with the health advice.

	DV: advice compliance			
	(1)	(2)	(3)	(4)
Sig other (vs. expert)	0.110*** (0.039)	0.159*** (0.036)	0.150*** (0.039)	0.161*** (0.041)
Demographics	NO	YES	YES	YES
Routine behaviors	NO	NO	YES	YES
Health	NO	NO	NO	YES
Constant	0.590*** (0.028)	−0.721*** (0.127)	−1.099*** (0.269)	−1.066*** (0.277)
Observations	605	605	605	605
R-squared	0.013	0.187	0.243	0.244

Robust standard errors in parentheses. ***p < 0.01, **p < 0.05, *p < 0.1.

To cross-validate the findings and address concerns about model choice, we also estimated logistic and ordered logistic regressions, reporting average marginal effects (see Supplementary Table S9). Table 7 presents the results across estimation strategies. Substantive conclusions remained consistent: single specified sources increased compliance by ~0.15, and significant others likewise by ~0.15. In the ordered logistic regression, we report the marginal effect of moving into the higher compliance categories (≥ 3 on the original 1–4 scale), allowing results to be directly compared with OLS and logit estimates.

**Table 7:** Comparison of marginal effect estimates across models (OLS, Logit, Ordered Logit).

Source contrast	OLS	Logit	Ordered logit
Single (vs. composite)	0.165*** (0.041)	0.149*** (0.037)	0.149*** (0.037)
Significant others (vs. experts)	0.161*** (0.041)	0.150*** (0.037)	0.150*** (0.037)

For logit and ordered logit models, reported values are average marginal effects (AMEs), which average predicted probability changes across all observations given their actual covariates, making them directly comparable to the percentage-point changes from OLS. Robust standard errors in parentheses. ***p < 0.01, **p < 0.05, *p < 0.1.

We further restricted the sample to participants who passed the manipulation checks for both source and message content. The effects of single specified sources (0.171, *p* < 0.01) and significant others (0.165, *p* < 0.01) remained significant and nearly identical (Columns 3 and 6 in Supplementary Table S8), confirming the robustness of our baseline results.

### 4.2 Mediation through cognitive mechanisms

We next examined whether the effects of source contrasts operated through cognitive pathways. Results are summarized in Table 8 (more details in Supplementary Table S10). For the contrast between single versus composite sources, the total effect on compliance was 0.166 (*p* < 0.01). A significant indirect pathway operated through credibility (0.036, *p* < 0.01), while attention, comprehension, and reactance did not transmit the effect. The remaining direct effect was 0.135 (*p* < 0.01). For the contrast between significant others versus experts, the total effect was 0.158 (*p* < 0.01). Two significant indirect pathways are shown: reduced reactance (0.069, *p* < 0.01) and increased credibility (0.027, *p* < 0.01). The direct effect was smaller at 0.068 (*p* = 0.07).

**Table 8:** Structural equation model estimation of the direct and indirect effects (via credibility and reactance) of information sources on intention to comply with health advice.

Comparison	Direct effect	Indirect via credibility	Indirect via reactance	Total effect
(1)	(2)	(3)	(4)	(5)
Single (vs. composite)	0.135*** (0.039)	0.036** (0.013)	−0.007 (n.s.) (0.009)	0.166*** (0.041)
Significant others (vs. experts)	0.068* (0.037)	0.027** (0.010)	0.069*** (0.017)	0.158*** (0.040)

More details are in Supplementary Table S10. Robust standard errors in parentheses. ***p < 0.01, **p < 0.05, *p < 0.1, n.s. = not significant.

As robustness checks, we estimated extended serial mediation models (e.g., source → attention → credibility/reactance → comprehension → compliance). None of the long sequential paths were statistically significant (Supplementary Tables S11, S12), supporting that the source effects operated through the immediate pathways of credibility and reactance.

### 4.3 Subgroup analysis

Audiences do not respond uniformly to health advice. Differences in health status, personal orientations, and socio-demographic background can shape how messages are interpreted and acted upon. Examining these patterns helps identify the boundary conditions of our findings and assess their broader generalizability. Although no specific hypotheses were developed, we explored heterogeneity across five domains: health conditions, health self-awareness, health literacy, behavioral alignment with the recommended practices, and demographics. These domains capture both objective and subjective factors known to influence health communication responses.

Health conditions capture vulnerability through the presence of chronic illness. Self-assessed healthiness, health concerns, and body image represent motivational orientations. Health literacy reflects the ability to access and evaluate health information. Behavioral alignment was measured by combining participants’ survey reports of daily routines with their assigned health message, capturing whether the advice matched existing habits. For instance, a participant who usually exercised in the morning was considered aligned when assigned the “morning exercise” message, and one whose first and last meals were typically within an eight-hour window was considered aligned when assigned the “16:8 fasting” message. Demographic factors such as age, gender, education, and income provide benchmarks for generalizability.

We estimated OLS models separately for each subgroup and applied Z-tests to formally assess whether coefficients differed across groups. Results are reported in Table 9. For the single versus composite contrast, effects were stronger among participants with chronic medical conditions (diff = 0.650, | *Z* | = 9.372, *p* < 0.01), higher health literacy through frequent health information access (diff = 0.151, | *Z* | = 1.696, *p* < 0.05), and behaviorally aligned routines (diff = 0.364, | *Z* | = 3.930, *p* < 0.01). For the significant others versus experts contrast, effects were stronger among participants with chronic conditions (diff = 0.366, | *Z* | = 4.763, *p* < 0.01), reporting body shame (diff = 0.247, | *Z* | = 2.803, *p* < 0.01), and behaviorally aligned routines (diff = 0.285, | *Z* | = 3.659, *p* < 0.01).

**Table 9:** OLS estimates on the information source effects on the intention to comply with the health advice across population groups.

Indep. Var.	Dimension	Variable	Yes/higher (than median)		No/lower (than median)		Diff (4)–(6)	Z-stat
			Coef.	Std. Err.	Coef.	Std. Err.		
(1)	(2)	(3)	(4)	(5)	(6)	(7)	(8)	(9)
Panel A: Single source (vs. composite source)	Health conditions	Overweight	0.156***	(0.058)	0.064	(0.064)	0.092	1.065
		Medical conditions	0.779***	(0.051)	0.129***	(0.047)	0.650***	9.372
	Health self-awareness	Perceived healthy of self	0.150***	(0.052)	0.210***	(0.053)	−0.060	−0.808
		Health concern of self	0.255***	(0.047)	0.270***	(0.076)	−0.015	−0.168
		Body shame	0.179***	(0.046)	−0.118	(0.086)	0.297***	3.045
	Health literacy	Health information access	0.269***	(0.075)	0.118**	(0.048)	0.151*	1.696
		Similar health advice	0.215***	(0.053)	0.114	(0.134)	0.101	0.701
	Routine behavior	Behavioral alignment	0.312***	(0.057)	−0.052	(0.073)	0.364***	3.930
	Demographics	Gender (female)	0.320***	(0.057)	0.172***	(0.045)	0.148**	2.038
		Age (below 30)	0.082	(0.055)	0.275***	(0.06)	−0.193**	−2.371
		Education (at least college)	0.130***	(0.048)	−0.054	(0.074)	0.184**	2.086
		Middle-class vs. low-income	−0.894***	(0.000)	0.165***	(0.044)	−1.059***	−24.062
		Perceived higher income	0.455***	(0.069)	0.05	(0.058)	0.405***	4.493
Panel B: Significant others (vs. experts)	Health conditions	Overweight	0.182***	(0.048)	0.125**	(0.053)	0.057	0.797
		Medical conditions	0.379***	(0.063)	0.013	(0.044)	0.366***	4.763
	Health self-awareness	Perceived healthy of self	0.069	(0.054)	0.629***	(0.056)	−0.560***	−7.198
		Health concern of self	0.073*	(0.042)	0.543***	(0.098)	−0.470***	−4.408
		Body shame	0.310***	(0.078)	0.063	(0.041)	0.247***	2.803
	Health literacy	Health information access	0.234***	(0.081)	0.083*	(0.046)	0.151	1.621
		Similar health advice	0.204***	(0.055)	0.061	(0.073)	0.143	1.565
	Routine behavior	Behavioral alignment	0.339***	(0.058)	0.054	(0.052)	0.285***	3.659
	Demographics	Gender (female)	0.333***	(0.053)	0.053	(0.061)	0.280***	3.465
		Age (below 30)	0.03	(0.062)	0.220***	(0.056)	−0.190**	−2.274
		Education (at least college)	0.098**	(0.045)	−0.043	(0.06)	0.141*	1.880
		Middle-class vs. low-income	−0.760***	(0.002)	0.175***	(0.042)	−0.935***	−22.237
		Perceived higher income	0.366***	(0.055)	0.019	(0.045)	0.347***	4.883

Robust standard errors in parentheses. ***p < 0.01, **p < 0.05, *p < 0.1.

Demographic differences were more modest. Women responded more strongly to significant others (diff = 0.280, | *Z* | = 3.465, *p* < 0.01), while older and more educated participants responded more strongly to single specified sources (diffs = 0.193 and 0.184; | *Z* | = 2.086 and 2.371, respectively). Income-based contrasts were also notable, with lower-income participants showing greater responsiveness to both source contrasts.

## 5 Discussion

### 5.1 Summary of findings

The findings provide clear answers to our research questions and the hypotheses derived from Source Credibility Theory (SCT) and the Persuasion Knowledge Model (PKM).

For H1 (the effects of source clarity), health messages attributed to single specified senders increased compliance by about 13–17 percentage points (p.p.) compared with composite diffuse sources. This finding supports H1a (i.e., the SCT prediction that clearly identifiable senders foster compliance through credibility) and does not support H1b (i.e., the PKM prediction that clearly identifiable sources trigger greater reactance). The magnitude of this effect appears substantial in public health contexts. For instance, Patel et al. (43) conducted a randomized trial of text-message reminders for COVID-19 vaccination and reported a 4.9 p.p. increase in adherence; Mehta et al. (44) suggested that a 5 p.p. gain can justify large-scale behavioral investment. Although these studies differ in context and outcome measures, making direct comparison less likely, they provide useful benchmarks for interpreting the magnitude of our findings. Viewed against these studies, the observed 13–17 p.p. increase highlights the practical significance of information source selection as a design lever in digital public health promotion.

For H2 (the effects of social proximity), messages from significant others increased compliance by approximately 11–16 p.p. compared with professional experts. This finding supports H2b (i.e., the PKM prediction that socially close messengers are more persuasive) while contradicting H2a (i.e., the SCT expectation that professional expertise would confer greater influence). In everyday self-care settings, relational authenticity appears to reduce resistance and enhance willingness to follow advice, outweighing the influence of formal expertise.

For H3 (cognitive mechanisms), the mediation analysis shows that the source clarity effect was partially transmitted by perceived credibility (≈ 4 p.p.), with no evidence of a reactance pathway. In contrast, the social proximity effect was explained by two reinforcing pathways: increased perceived credibility (≈ 3 p.p.) and reduced psychological reactance (≈ 7 p.p.). This supports both H3a and H3b in the proximity contrast, indicating that socially close senders gain influence by simultaneously boosting credibility and lowering reactance. Attention and comprehension did not significantly mediate the main effects, likely because these variables capture how participants processed message *content* rather than how they evaluated the *source*. As message content was identical across conditions, little variation in these measures was expected. By contrast, credibility and reactance, central to SCT and PKM, are inherently source-sensitive, explaining why they accounted for the observed effects. These results suggest that in rapid, feed-based exposures, judgments about *who* is speaking dominate over deeper processing of *what* is said.

Finally, exploratory subgroup analyses highlight important boundary conditions. Source effects were stronger among participants who found health information personally salient (e.g., those with chronic conditions or body-image concerns) and among those whose routine behaviors already aligned with the recommended practices. These patterns suggest that the general advantages of identifiable and socially close sources may be amplified when health information resonates with individuals’ existing concerns and habits. While the overall findings are robust, their generalizability should be interpreted with these boundary conditions in mind.

### 5.2 Contributions to theory and literature

This study advances theory in three ways. First, we refine Source Credibility Theory (SCT) for contemporary, repost-driven digital environments by showing that source clarity (i.e., whether a sender is perceived as clearly identifiable) is a prerequisite for credibility to operate. Messages attributed to identifiable senders were more persuasive than those from composite or diffuse sources, and this advantage was transmitted mainly through perceived credibility. This finding extends SCT beyond its traditional focus on expertise to emphasize that credibility depends not only on what a source knows but also on who the audience perceives as speaking. It aligns with prior work showing that visible author cues enhance credibility (6–8) and challenges the assumption that aggregated or richly detailed content guarantees trust when the speaker is indistinct.

Second, we clarify when the Persuasion Knowledge Model (PKM) applies. Within the class of single specified senders, social proximity proved decisive: advice from significant others outperformed that from professional experts, operating through two reinforcing pathways: higher credibility and lower reactance. While PKM anticipates reactance when persuasive intent is salient, our findings show that relational authenticity characteristic of close ties can reduce such resistance and enhance credibility, even without formal expertise. This aligns with diffusion and network research showing that proximate messengers can effectively promote health behaviors (11, 22) and extends crisis-communication studies where expert voices often dominate (12, 45, 46). The theoretical implication is not that experts fail to persuade, but that PKM helps explain why professional authority can elicit reactance in everyday, low-stakes contexts, whereas advice from close ties tends to be perceived as genuine and acceptable.

Third, we contribute to scholarship on cognitive pathways in digital health communication. Under brief, feed-style exposures, source-level cues (who is speaking) outweighed message-level processing (attention, comprehension) in explaining compliance. Although dual-process models imply that deeper processing matters under high involvement, our mediation analysis indicates that in rapid, everyday encounters, credibility and reactance, not attention or content comprehension, drive behavioral intentions. This points to boundary conditions: in short, familiar formats, sender cues dominate, whereas in longer, higher-stakes settings, content processing may regain importance. Future theorizing on public health communication should therefore treat source clarity and social proximity as key contextual factors shaping which cognitive routes influence compliance.

Collectively, these contributions integrate SCT and PKM rather than privileging one framework: SCT better explains the clarity contrast (via credibility), whereas PKM better explains the proximity contrast (via reduced reactance alongside credibility). By adapting classic theories to composite and socially filtered digital environments, this study offers a parsimonious framework for predicting when identifiable and socially close sources are most persuasive in digital public health promotion.

### 5.3 Implications for practice

The findings offer practical guidance for digital health communication, online content design, and public health interventions. They show that persuasiveness depends less on the quantity of information and more on who appears to be speaking and how messages are framed.

For individuals and their peer networks, health advice is more likely to be adopted when it comes from trusted and clearly identifiable sources such as family members, friends, or familiar professionals. Campaigns can encourage reliance on identifiable senders rather than anonymous or aggregated outputs. Peer-support initiatives like Weight Watchers illustrate how social accountability and encouragement from known others can sustain behavior change. At the same time, individuals should verify the scientific accuracy of advice, especially for complex health issues where relational trust cannot replace medical expertise.

For digital platforms, our findings highlight credibility and reactance as key design levers. Platforms like X (formerly Twitter), TikTok, or Xiaohongshu can strengthen perceived credibility by making sender identities visible and verifiable, while reducing perceived pressure through balanced framing and recommendation algorithms. For example, Xiaohongshu’s health communities show how everyday sharing can motivate healthy habits, provided these spaces are accompanied by moderation policies that prevent persuasive but inaccurate content from spreading and eroding public trust.

For public campaigns and media organizations, authenticity and emotional resonance are critical for reducing resistance. Campaigns that feature family members, friends, or relatable community figures tend to be more appealing and less likely to provoke reactance, whereas authoritative expert messages that lack personal touch can face skepticism. NHS England’s use of real-life patient stories demonstrates how narratives rooted in lived experience can expand the reach and acceptance of health messages. Similarly, media outlets can pair credible information with personalized storytelling rather than relying solely on institutional authority.

For health authorities, the challenge is to balance professionalism with approachability. Technical accuracy remains essential, but overly clinical or directive communication may trigger defensiveness. A more effective approach combines expert guidance with relatable narratives, especially in chronic disease management, where patients need both accurate information and sustained encouragement. Blending medical authority with community or peer voices can simultaneously enhance credibility and lower reactance, improving the overall effectiveness of digital public health communication.

### 5.4 Limitations and future research

Despite its contributions, this study has several limitations. First, although the randomized experimental design is robust, it was implemented in a general social media context. Replication in specialized settings such as telehealth platforms or disease-specific communities would clarify how platform features shape credibility and behavioral responses.

Second, the crowd-sourced sample, while diverse, may not represent older or culturally distinct populations. Targeted and cross-cultural studies could examine how age, culture, and health literacy moderate source effects. Moreover, as this experiment focused on weight management, future research should test whether similar mechanisms operate in other domains such as mental health, medication adherence, or chronic disease management.

Third, compliance intentions rather than actual behaviors were measured. Longitudinal and behavioral data are needed to assess whether source effects persist in real-world contexts. Our classification also grouped Wikipedia and AI chatbots as composite sources, though users may perceive these differently when transparency varies; this distinction warrants future testing.

A further limitation concerns the measurement of the two source dimensions. The source-and-topic awareness questions (described in Section 3.2.4) only confirmed that participants noticed the assigned source and message content. However, they did not test whether the manipulations changed perceptions of source clarity or social proximity. Although the experiment varied source labels, it did not directly ask how participants feel about each source as identifiable or socially close. Future studies should include direct measures or crossed manipulations of these dimensions to validate their effects on health advice compliance.

Finally, the study examined static online messages, whereas interactive AI tools and personalized recommendation systems are becoming central gatekeepers of health information. Future work should refine the measurement of mediators in these richer environments. In our study, credibility and reactance were assessed by self-report; behavioral indicators of trust or resistance could complement survey measures. Moreover, while attention and comprehension were not key mediators here, they may become more relevant in multimodal or interactive health communication contexts.

## 6 Concluding remarks

This study provides experimental evidence that source clarity and social proximity are central determinants of compliance with online health advice. Messages from single specified senders, especially significant others, increased compliance intentions compared with composite diffuse sources or professional experts. Mediation analysis showed that these effects operated primarily through perceived credibility and, in the case of significant others, also through reduced psychological reactance, whereas attention and comprehension did not function as operative pathways in brief, feed-like encounters. Effects were particularly pronounced among participants with chronic conditions, heightened body-image concerns, higher health literacy, or routine behaviors aligned with the advice, underscoring boundary conditions for generalizability. The findings challenge the assumption that professional authority or information aggregation alone ensures acceptance, highlighting instead that identifiable provenance, relational authenticity, and credibility judgments drive compliance in everyday digital contexts. As public health communication increasingly depends on social platforms and AI intermediaries, embedding sources that are both clearly attributable and socially resonant will be essential for translating information exposure into meaningful public health action. Future research should extend these insights to diverse populations, alternative health domains, and interactive or multimodal environments. Advancing this agenda will require collaboration between researchers, practitioners, and platform designers to ensure digital health communication is both scientifically sound and socially effective.

## Data Availability

The raw data supporting the conclusions of this article will be made available by the authors, without undue reservation.

## Footnotes

^1^ https://shorturl.at/jFxyI

^2^ https://shorturl.at/fzeOJ
