# Supplementary material for: Who do we follow online? An experimental study on source clarity and social proximity in digital health communication
Source: Front Public Health. 2025 Oct 27;13:1661328. doi: 10.3389/fpubh.2025.1661328 (PMC12597950; doi:10.3389/fpubh.2025.1661328)
Supplement: Supplementary file 1 [file Data_Sheet_1.PDF]

Supplementary Material

TABLE A-1. Definitions and measurements of key variables

Variable Name	Description	Measurements & Survey Items
Information source	Treatment variable indicating the attributed sender of the health advice.	Eight labels: parent, friend, work colleague, doctor, health influencer, news agency, Wikipedia, AI chatbot. Analysis contrasts: (i) Single specified (= 1) vs. Composite diffuse (= 0); (ii) Within single source, Significant others (= 1) vs. Professionals (= 0).
Compliance intention	Behavioral tendency outcome: likelihood of following the health advice.	Participants indicated compliance intention on a four-point ordinal scale: (1) "*I will not take the advice*"; (2) "*I will consider the advice but lean toward not taking it*"; (3) "*I will consider the advice and lean toward taking it*"; and (4) "*I will take the advice*". For the main analyses, we dichotomized the measure by combining responses (1–2) versus (3–4), coding willingness to comply as 1 and non-compliance as 0. As robustness checks, we also estimated ordered logit models using the full four-point scale.
Perceived Expertise	Source credibility dimension: expertise	"The source is knowledgeable about the topic."<br>"The source has expertise in this area."<br>"The source is well-informed."
Perceived Trustworthiness	Source credibility dimension: trustworthiness	"The source is honest."<br>"The source is trustworthy"<br>"I believe what the source says"
Perceived Attractiveness	Source credibility dimension: attractiveness	"The source is likable."<br>"The source is attractive"<br>"The source is appealing"
Reactance	Resistance or perception of manipulation	"The health message tries to manipulate me."<br>"I feel like my freedom to choose is being restricted."<br>"I want to do the opposite of what it recommends."<br>"The message makes me feel defensive"<br>"I am less likely to do what the message suggests"<br>"I want to do the opposite of what the message recommends"
Attention	Depth of engagement with the health message	"I paid close attention to the health information."<br>"I was fully focused while reading the health content"<br>"The health information captured my interest"<br>"I found the health information engaging"
Comprehension	Objective recall/understanding of the health post	Five multiple-choice factual questions about the assigned health message. For example, for the participants assigned to "16:8 Fasting is Good for Your Health," the reading comprehension questions are the following:<br>"1. What is the main idea of the 16:8 diet?"<br>"2. What benefit does the 16:8 diet offer in terms of calorie intake?"<br>"3. How does the 16:8 diet help with fat burning?"<br>"4. Which of the following is NOT a benefit of the 16:8 diet mentioned in the passage?"<br>"5. What is the process called that aids in cell repair during the 16:8 fasting period?"<br>Score = proportion correct (0–1).

Note: For credibility, reactance, and attention measures, 7-point agreement ratings are used (–3 = Strongly disagree to +3 = Strongly agree). For each variable, Composite averaged across items is used as the measurement.

TABLE A-2. Definitions and measurements of control variables

Variable Name	Description	Survey Question & Response Options
Gender	Gender of the participant	“What is your gender?” (1 = Male, 2 = Female)
Age	Age in years	“What is your age?” (Open numeric entry)
Education	Highest education level	“What is your highest completed level of education?” (1 = No formal schooling, 2 = Primary, 3 = Secondary, 4 = High school, 5 = Bachelor’s, 6 = Master’s, 7 = Doctorate)
Income	Household income (categorical)	“What is your household’s approximate annual income?” (12 brackets from “< $10,000” to “> $100,000”)
Perceived Income	Self-evaluation of financial situation	“How do you evaluate your family’s income compared to others?” (1 = Much lower, 2 = Lower, 3 = Higher, 4 = Much higher)
BMI	Body Mass Index	Computed using self-reported height and weight
Alcohol	Frequency of alcohol consumption	“How often do you drink alcohol?” (0 = Never, 1 = Monthly, 2 = Weekly, 3 = Several times a week, 4 = Daily)
Smoke	Smoking status	“Do you smoke?” (0 = No, 1 = Yes)
Medical Conditions	Presence of chronic medical conditions	“Do you currently have any chronic medical conditions?” (0 = No, 1 = Yes)
Self-perceived Healthiness	Self-assessed health status	“How healthy do you think you are?” (–3 = Very unhealthy to +3 = Very healthy)
Health Concern	Level of concern about health	“How concerned are you about your health?” (–3 = Not at all concerned to +3 = Very concerned)
Body Shame	Body image concern	“Do you feel ashamed of your body shape/weight?” (–3 = Strongly disagree to +3 = Strongly agree)
Health Info Frequency	Frequency of health information search	“How often do you look for health-related information online?” (0 = Never to 6 = Several times per day)
Daily Routines (timing of meal or exercise)	Lifestyle controls	“What time do you usually have breakfast/lunch/dinner?” (0–24 hour clock).
Daily Routines (Duration of work or leisure)	Lifestyle controls	“How many hours do you usually spend sleeping / sitting for work / sitting for leisure / exercising per day?” (Numeric entry)

TABLE A-3. Balance check of covariates (composite vs. single source)

Control variables	Composite		Single		Comparison	
	(1)	(2)	(3)	(4)	(5)	
	Mean	Std. Dev.	Mean	Std. Dev.	Difference in mean	
Gender	1.393	(0.456)	1.446	(0.498)	0.054	(0.089)
Age	29.317	(9.345)	28.992	(8.798)	-0.325	(1.623)
Education	4.146	(1.478)	4.388	(1.352)	0.242	(0.251)
Income	3.195	(2.846)	2.967	(2.421)	-0.228	(0.460)
Income (perceived)	2.122	(0.671)	2.182	(0.772)	0.060	(0.136)
Health info frequency	1.861	(1.453)	2.198	(1.569)	0.337	(0.280)
breakfast	9.024	(2.671)	8.876	(1.867)	-0.148	(0.381)
Timing for breakfast	13.561	(2.657)	13.777	(2.112)	0.216	(0.411)
Timing for lunch	19.537	(1.800)	19.686	(2.352)	0.149	(0.404)
Timing for dinner	15.000	(5.140)	15.116	(5.007)	0.116	(0.915)
Timing for exercise	7.202	(0.940)	7.021	(1.655)	-0.182	(0.274)
Duration of sleeping	6.083	(2.868)	6.390	(2.837)	0.307	(0.517)
Duration of sitting for work	4.178	(2.802)	4.106	(2.403)	-0.072	(0.456)
Duration of sitting for leisure	1.158	(0.905)	1.204	(0.760)	0.045	(0.154)
Alcohol (0-4)	1.317	(1.241)	1.397	(1.065)	0.080	(0.202)
Smoke (0-1)	0.244	(0.430)	0.223	(0.417)	-0.021	(0.076)
Self-perceived healthiness	-0.073	(1.763)	0.165	(1.562)	0.238	(0.293)
Self-health concern	0.537	(1.437)	0.521	(1.592)	-0.016	(0.282)
Self-body shame	0.829	(1.484)	0.711	(1.699)	-0.119	(0.299)
Medical conditions (0-1)	0.171	(0.377)	0.289	(0.454)	0.119	(0.079)
Similar advice heard	0.561	(0.497)	0.554	(0.498)	-0.007	(0.090)
Similar advice followed	0.478	(0.502)	0.493	(0.501)	0.014	(0.122)

Notes: We use t-statistics to test the differences across groups (in Column 5), and none are statistically significant, indicating that the randomization was well performed. Standard deviations are in the parentheses.

TABLE A-4. Balance check of covariates (professional expert vs. significant others)

Control variables	Professional Expert		Significant Other		Comparison	
	(1)	(2)	(3)	(4)	(5)	
	Mean	Std. Dev.	Mean	Std. Dev.	Differences in mean	
Gender	1.443	(0.498)	1.450	(0.498)	0.007	(0.091)
Age	30.541	(8.947)	27.417	(8.369)	-3.124*	(1.586)
Education	4.492	(1.400)	4.283	(1.294)	-0.208	(0.247)
Income	3.246	(2.417)	2.683	(2.395)	-0.563	(0.440)
Income (perceived)	2.230	(0.688)	2.133	(0.847)	-0.096	(0.141)
Health info frequency	2.197	(1.620)	2.200	(1.517)	0.003	(0.287)
breakfast	8.639	(1.711)	9.117	(1.987)	0.477	(0.339)
Timing for breakfast	13.738	(1.875)	13.817	(2.331)	0.079	(0.387)
Timing for lunch	19.525	(2.848)	19.850	(1.694)	0.325	(0.430)
Timing for dinner	15.180	(5.183)	15.050	(4.830)	-0.130	(0.917)
Timing for exercise	6.915	(1.300)	7.128	(1.948)	0.214	(0.303)
Duration of sleeping	6.289	(2.468)	6.493	(3.170)	0.205	(0.519)
Duration of sitting for work	3.648	(1.970)	4.572	(2.699)	0.924**	(0.432)
Duration of sitting for leisure	1.107	(0.644)	1.298	(0.848)	0.191	(0.144)
Alcohol (0-4)	1.475	(1.097)	1.317	(1.026)	-0.159	(0.195)
Smoke (0-1)	0.213	(0.410)	0.233	(0.424)	0.020	(0.076)
Self-perceived healthiness	0.361	(1.539)	-0.033	(1.562)	-0.394	(0.284)
Self-health concern	0.902	(1.413)	0.133	(1.671)	-0.768***	(0.283)
Self-body shame	0.902	(1.699)	0.517	(1.681)	-0.385	(0.309)
Medical conditions (0-1)	0.328	(0.470)	0.250	(0.434)	-0.078	(0.083)
Similar advice heard	0.557	(0.498)	0.550	(0.498)	-0.007	(0.091)
Similar advice followed	0.382	(0.487)	0.606	(0.490)	0.224*	(0.121)

Notes: We use t-statistics to test the differences across groups (in Column 5), and none are statistically significant, indicating that the randomization was well performed. Standard deviations are in the parentheses.

TABLE A-5. Balance check of covariates (across all eight source groups)

Control Variables	(1) Patient	(2) Friend	(3) Colleague	(4) Doctor	(5) Health Influencer	(6) News agency	(7) Wikipedia	(8) AI Chatbot	(9) Pearson χ^2	(10) p-values
Gender	1.368	1.476	1.500	1.524	1.421	1.381	1.333	1.250	5.0253	0.657
	(0.496)	(0.512)	(0.513)	(0.512)	(0.507)	(0.498)	(0.483)	(0.444)		
Age	27.737	26.381	28.200	28.619	33.421	29.857	30.286	28.300	261.316	0.226
	(8.912)	(8.158)	(8.557)	(6.415)	(12.167)	(7.532)	(11.516)	(6.768)		
Education	4.263	4.095	4.500	4.667	4.368	4.429	4.048	4.250	42.851	0.435
	(1.447)	(1.338)	(1.147)	(1.560)	(1.342)	(1.363)	(1.396)	(1.618)		
Income	2.421	3.286	2.300	2.619	3.737	3.429	3.429	2.950	71.359	0.66
	(2.364)	(3.101)	(1.418)	(1.499)	(3.142)	(2.441)	(3.487)	(2.114)		
Income (perceived)	2.316	2.095	2.000	2.286	2.263	2.143	2.143	2.100	17.695	0.668
	(1.003)	(0.831)	(0.725)	(0.717)	(0.653)	(0.727)	(0.655)	(0.718)		
Health info frequency	2.316	2.048	2.250	2.048	2.000	2.524	1.190	1.950	37.929	0.650
	(1.376)	(1.717)	(1.517)	(1.431)	(1.732)	(1.750)	(1.250)	(1.605)		
Timing for breakfast	9.000	9.238	9.100	8.524	8.263	9.095	9.000	9.050	70.241	0.469
	(1.732)	(1.640)	(2.594)	(1.327)	(1.485)	(2.189)	(2.588)	(2.874)		
Timing for lunch	14.211	13.952	13.300	13.619	13.526	14.048	13.143	14.000	74.320	0.565
	(1.437)	(1.359)	(3.570)	(1.532)	(1.219)	(2.617)	(3.260)	(1.892)		
Timing for dinner	20.158	20.095	19.300)	19.571	19.842	19.190	19.190	19.900	75.687	0.521
	(2.115)	(1.179)	(1.689)	(1.859)	(2.007)	(4.167)	(1.750)	(1.861)		
Timing for exercise	14.053	15.238	15.800	15.143	15.158	15.238	15.714	14.250	127.729	0.763
	(4.720)	(5.338)	(4.549)	(6.126)	(4.375)	(5.186)	(5.139)	(5.270)		
Duration of sleeping	7.353	6.738	7.325	6.643	6.842	7.252	7.390	7.620	273.725	0.359
	(1.602)	(2.208)	(2.031)	(1.835)	(0.679)	(1.071)	(0.897)	(1.011)		
Duration of sitting for work	6.237	7.105	6.095	5.990	6.300	6.576	5.948	6.225	312.030	0.425
	(3.877)	(2.419)	(3.254)	(2.735)	(2.305)	(2.464)	(3.018)	(2.835)		
Duration of sitting for leisure	4.174	4.348	5.185	3.348	4.005	3.624	5.457	4.270	337.955	0.460
	(2.172)	(1.924)	(3.735)	(1.514)	(2.563)	(1.840)	(3.224)	(2.271)		
Alcohol (0-4)	1.632	1.286	1.050	1.571	1.474	1.381	1.476	1.150	24.145	0.674
	(1.212)	(1.102)	(0.686)	(1.076)	(1.073)	(1.203)	(1.436)	(1.040)		
Smoke (0-1)	0.105	0.333	0.250	0.143	0.158	0.333	0.333	0.150	7.729	0.357
	(0.315)	(0.483)	(0.444)	(0.359)	(0.375)	(0.483)	(0.483)	(0.366)		
Self-perceived healthiness	-0.316	0.190	0.000	0.762	0.158	0.143	-0.190	0.050	52.995	0.119
	(1.336)	(1.721)	(1.654)	(1.546)	(1.119)	(1.852)	(1.778)	(1.820)		
Self-health concern	0.000	0.190	0.200	0.714	1.105	0.905	0.714	0.350	56.147	0.071
	(1.333)	(2.089)	(1.576)	(1.554)	(1.150)	(1.546)	(1.309)	(1.599)		
Self-body shame	0.368	0.571	0.600	1.190	0.947	0.571	0.619	1.050	43.229	0.419
	(1.739)	(1.886)	(1.501)	(1.806)	(1.393)	(1.886)	(1.564)	(1.432)		
Medical conditions (0-1)	0.263	0.333	0.150	0.333	0.263	0.381	0.143	0.200	5.913	0.550
	(0.452)	(0.483)	(0.366)	(0.483)	(0.452)	(0.498)	(0.359)	(0.410)		
Similar advice heard	0.684	0.571	0.400	0.571	0.526	0.571	0.619	0.500	3.957	0.785
	(0.478)	(0.507)	(0.503)	(0.507)	(0.513)	(0.507)	(0.498)	(0.513)		
Similar advice followed	0.615	0.667	0.500	0.417	0.200	0.500	0.462	0.500	5.994	0.540
	(0.506)	(0.492)	(0.535)	(0.515)	(0.422)	(0.522)	(0.519)	(0.527)		

Notes: Standard deviations are in the parentheses. Covariate balance across eight groups is tested with Pearson's χ^2; p-values in Column 10 show no imbalance, indicating successful randomization.

TABLE A-6. Design of health messages

Panel A: Health message about healthy eating
16:8 Fasting is Good for your Health<br><br>The 16:8 diet involves fasting for 16 hours and eating within an 8-hour window each day. This method can effectively reduce calorie intake, promote fat burning, and is easier to maintain since it doesn't require completely cutting out any foods. Additionally, the long fasting period can help the body enter a state of autophagy, which aids in cell repair and anti-aging.
Panel B: Health message about healthy eating
16:8 Fasting is Not Good for Your Health<br><br>Long-term adherence to the 16:8 diet could lead to malnutrition, especially from inadequate protein and vitamin intake. The 16-hour fasting period may cause persistent hunger, dizziness, and binge eating, which can disrupt metabolism and affect physical performance. Over time, this could also strain nutritional balance and overall energy levels, potentially causing long-term health issues.
Panel C: Health message about exercise
Morning Exercise is Good for Your Health<br><br>Metabolism is most active in the morning, making exercise more effective for fat loss and helping to kickstart your day. Morning workouts boost your metabolism, enhance energy levels, and improve mood. Exercising at night, however, can interfere with your body's natural winding-down process, potentially disrupting your circadian rhythm and leading to insomnia or poor sleep quality.
Panel D: Health message about exercise
Evening Exercise is Good for Your Health<br><br>Exercising Immediately after waking up may lead to low blood sugar levels and increase the risk of cardiovascular issues. In contrast, physical performance tends to peak in the late afternoon and evening, when your body is fully awake and nourished. This time is optimal for muscle building and can enhance strength and endurance, as your body is more prepared for intense physical activity.

TABLE A-7. Manipulation Checks

Check	Pass Rate (%)
Source manipulation	91.358
Measure topic manipulation	82.716
Both	75.310

TABLE A-8. Robustness checks after excluding participants not passing manipulation checks

DV: Compliance	Panel A: Effect of single source (versus composite source)			Panel B: Effect of significant others (versus professional experts)		
	(1)	(2)	(3)	(4)	(5)	(6)
Single Source (vs. Composite Source)	0.166***	0.173***	0.171***			
	(0.042)	(0.043)	(0.068)			
Sig Other (vs. Expert)				0.158***	0.167***	0.165***
				(0.041)	(0.042)	(0.043)
Demographics	YES	YES	YES	YES	YES	YES
Routine behaviors	YES	YES	YES	YES	YES	YES
Health	YES	YES	YES	YES	YES	YES
Constant	-0.080	-0.040	-0.151	-0.789***	-0.891***	-1.238***
	(0.253)	(0.272)	(0.287)	(0.264)	(0.286)	(0.281)
Observations	735	670	560	555	500	475
R-squared	0.202	0.212	0.223	0.236	0.260	0.302

Notes: Columns 1 and 3 are original estimates for the effect of single source (versus composite source) and the effect of significant others (versus professionals), respectively, but including participants who have not passed the manipulation checks. Columns 2 and 5 exclude those who did not pass the manipulation checks for information sources, and Columns 3 and Column 6 exclude those who did not pass both manipulation checks for information sources and health message content. All covariates including demographics, routine behaviors, and health-related variables are included in the analysis. Robust standard errors in parentheses. *** p<0.01, ** p<0.05, * p<0.1

TABLE A-9. Logistic and ordered logistic regression estimation of the information source effects

DV: Compliance intention	Panel A: Logit		Panel B: Ordered Logit	
	(1)	(2)	(3)	(4)
Single Source (vs. Composite Source)	0.813***		0.813***	
	(0.207)		(0.207)	
Sig Other (vs. Expert)		0.905		0.905***
		(0.234)		(0.234)
Demographics	YES	YES	YES	YES
Routine behaviors	YES	YES	YES	YES
Health	YES	YES	YES	YES
Constant	-4.589***	-9.620***	4.589***	9.620***
	(1.409)	(1.756)	(1.409)	(1.756)
Marginal Effects (dy/dx)	0.149***	0.150***	0.149***	0.150***
	(0.037)	(0.037)	(0.037)	(0.037)
Observations	735	735	735	735

Notes: For both models, reported values in the shaded cells are average marginal effects (AMEs), which average predicted probability changes across all observations given their actual covariates, making them directly comparable to the percentage-point changes from OLS. Compliance intention was measured by response to a four-point ordinal scale: (1) “I will not take the advice”; (2) “I will consider the advice but lean toward not taking it”; (3) “I will consider the advice and lean toward taking it”; and (4) “I will take the advice”. For OLS and logit analyses, we dichotomized the measure by combining responses (1–2) versus (3–4), coding willingness to comply as 1 and non-compliance as 0. Ordered logit models using the full four-point scale, comparing the likelihood of being in higher compliance categories (≥3) versus lower ones (<3). Robust standard errors in parentheses. *** p<0.01, ** p<0.05, * p<0.1

TABLE A-10. Structural equation model with simultaneous mediators to test Hypothesis 3 (Source → Credibility/Reactance/Attention/Comprehension → Compliance)

	Panel A: Effect of single source (versus composite source)		Panel B: Effect of sig others (versus professional experts)	
Pathway	Coef.	Std. Err.	Coef.	Std. Err.
Source → Attention	–0.276***	0.068	0.127*	0.067
Attention → Credibility	0.129***	0.015	0.028*	0.012
Attention → Reactance	0.088	0.109	–0.599***	0.089
Attention → Comprehension	0.309**	0.110	0.302**	0.102
Credibility → Comprehension	0.016	0.022	–0.009	0.028
Reactance → Comprehension	0.054	0.109	–0.154	0.131
Compliance ← Credibility	–0.082***	0.015	–0.115***	0.018
Compliance ← Reactance	0.116***	0.015	0.088***	0.020
Compliance ← Attention	0.135***	0.039	0.068*	0.037
Compliance ← Comprehension	–0.004	0.006	–0.001	0.003
**Direct:** Source → Compliance	0.007	0.014	–0.004	0.004
**Indirect:** Source → Credibility → Compliance	0.036**	0.013	0.027**	0.010
**Indirect:** Source → Reactance → Compliance	–0.007	0.009	0.069***	0.017
**Indirect:** Source → Attention → Compliance	–0.004	0.006	–0.001	0.003
**Indirect:** Source → Comprehension → Compliance	0.007	0.014	–0.004	0.004
**Total Effect**	0.166***	0.041	0.158***	0.040

Notes: To clarify whether the effects of information sources on compliance operate through intermediate cognitive responses, we estimated a structural equation model with bootstrapped standard errors (100 replications). In this model, compliance (binary) was modeled as a linear-probability outcome regressed on the experimental source treatment and four mediators in parallel: perceived credibility, psychological reactance, attention, and comprehension. Perceived credibility was modeled as a latent construct combining expertise, trustworthiness, and attractiveness. This specification allows each cognitive response to transmit source effects to compliance, while retaining a direct path from source to compliance. The model includes demographics, lifestyle, and health-related control variables, which are omitted here for brevity. The coefficients for direct and indirect effects in the shaded cells above are also reported in Table 8 of the manuscript. *** $p<0.01$, ** $p<0.05$, * $p<0.1$.

TABLE A-11. Robustness check using an alternative structural equation model with serial mediators (Source → Attention → Credibility/Reactance → Comprehension → Compliance)

	Panel A: Effect of single source (versus composite source)		Panel B: Effect of sig others (versus professional experts)	
Pathway	Coef.	Std. Err.	Coef.	Std. Err.
Source → Attention	–0.276***	0.068	0.127*	0.067
Attention → Credibility	0.460***	0.048	0.545***	0.059
Attention → Reactance	–0.601***	0.052	–0.587***	0.062
Attention → Comprehension	0.014*	0.008	0.022**	0.008
Credibility → Comprehension	–0.028***	0.006	–0.039***	0.006
Reactance → Comprehension	–0.013*	0.008	–0.023**	0.007
Compliance ← Credibility	0.116***	0.015	0.088***	0.020
Compliance ← Reactance	–0.082***	0.015	–0.115***	0.018
Compliance ← Attention	0.016	0.022	–0.009	0.028
Compliance ← Comprehension	0.054	0.109	–0.154	0.131
**Direct:** Source → Compliance	0.135***	0.039	0.068*	0.037
**Indirect:** Source → Attention → Credibility → Comprehension → Compliance	0.00019	0.00038	0.00041	0.00040
**Indirect:** Source → Attention → Reactance→ Comprehension → Compliance	–0.00012	0.00024	–0.00027	0.00026
**Total Effect**	0.135***	0.039	0.068*	0.037

Notes: To further test other potential pathways through which information sources affect compliance, we estimated an alternative structural equation model with bootstrapped standard errors (100 replications). In this model, cognitive factors are following a longer chain than that in Table A-10: Source → Attention → Credibility/Reactance → Comprehension → Compliance. Perceived credibility was modeled as a latent construct combining expertise, trustworthiness, and attractiveness. The model includes demographics, lifestyle, and health-related control variables, which are omitted here for brevity. *** p<0.01, ** p<0.05, * p<0.1.

TABLE A-12. Robustness check using an alternative structural equation model with serial mediators (Source → Credibility/Reactance → Attention → Comprehension → Compliance)

	Panel A: Effect of single source (versus composite source)		Panel B: Effect of sig others (versus professional experts)	
Pathway	Coef.	Std. Err.	Coef.	Std. Err.
Source → Credibility	0.309**	0.110	0.302**	0.102
Source → Reactance	0.088	0.109	–0.599***	0.089
Credibility → Attention	0.088***	0.020	0.129***	0.033
Reactance → Attention	–0.202***	0.024	–0.188***	0.032
Source → Attention	–0.286***	0.061	–0.025	0.062
Attention → Comprehension	0.030***	0.008	0.023**	0.008
Credibility → Comprehension	–0.037***	0.005	–0.039***	0.006
Reactance → Comprehension	–0.016*	0.007	–0.020**	0.007
Compliance ← Credibility	0.116***	0.015	0.088***	0.020
Compliance ← Reactance	–0.082***	0.015	–0.115***	0.018
Compliance ← Attention	0.016	0.022	–0.009	0.028
Compliance ← Comprehension	0.054	0.109	–0.154	0.131
**Direct:** Source → Compliance	0.135***	0.039	0.068*	0.037
**Indirect:** Source → Credibility → Attention → Comprehension → Compliance	0.00004	0.00009	–0.00013	0.00014
**Indirect:** Source → Reactance → Attention → Comprehension → Compliance	–0.00003	0.00007	–0.00039	0.00038
**Total Effect**	0.135***	0.039	0.067*	0.037

Notes: To further test other potential pathways through which information sources affect compliance, we estimated an alternative structural equation model with bootstrapped standard errors (100 replications). In this model, cognitive factors are following a longer chain than that in Table A-10: Source → Credibility/Reactance → Attention → Comprehension → Compliance. Perceived credibility was modeled as a latent construct combining expertise, trustworthiness, and attractiveness. The model includes demographics, lifestyle, and health-related control variables, which are omitted here for brevity. *** p<0.01, ** p<0.05, * p<0.1.
